# Inhibitory Effects of Saponin-Rich Extracts from *Pouteria cambodiana* against Digestive Enzymes α-Glucosidase and Pancreatic Lipase

**DOI:** 10.3390/foods12203738

**Published:** 2023-10-11

**Authors:** Kawisara Sanneur, Noppol Leksawasdi, Nutsuda Sumonsiri, Charin Techapun, Siraphat Taesuwan, Rojarej Nunta, Julaluk Khemacheewakul

**Affiliations:** 1 Division of Food Science and Technology, School of Agro-Industry, Faculty of Agro-Industry, Chiang Mai University, Chiang Mai 50100, Thailand; kawisara_sa@cmu.ac.th (K.S.); siraphat.t@cmu.ac.th (S.T.); 2 Bioprocess Research Cluster, School of Agro-Industry, Faculty of Agro-Industry, Chiang Mai University, Chiang Mai 50100, Thailand; noppol.l@cmu.ac.th (N.L.); charin.t@cmu.ac.th (C.T.); quan_rn@hotmail.com (R.N.); 3 The Cluster of Agro Bio-Circular-Green Industry (Agro BCG), Faculty of Agro-Industry, Chiang Mai University, Chiang Mai 50100, Thailand; 4 School of Health and Life Sciences, Teesside University, Middlesbrough TS1 3BX, UK; n.sumonsiri@tees.ac.uk; 5 Division of Food Science and Technology, Faculty of Science and Technology, Lampang Rajabhat University, Lampang 52100, Thailand

**Keywords:** *Pouteria cambodiana*, bark, saponin, α-glucosidase, pancreatic lipase, ultrasound

## Abstract

*Pouteria cambodiana* is a perennial plant that has a wide distribution in tropical regions. It is commonly referred to as ’Nom-nang’ in the northern region of Thailand. The bark of this plant has been used for the purpose of promoting lactation among breastfeeding mothers. Moreover, *P. cambodiana* bark has a high nutraceutical potential due to the presence of saponins, which are secondary metabolites. The purpose of this study was to determine the optimal conditions for ultrasound-assisted extraction (UAE) of saponins from the bark of *P. cambodiana* and to assess the in vitro inhibitory activities of saponin-rich extracts. The most effective extraction conditions involved a temperature of 50 °C and a 50% concentration level of ethanol as the solvent, which allowed the extraction of saponin at a concentration of 36.04 mg/g. Saponin-rich extracts and their hydrolysates from *P. cambodiana* bark were evaluated for their ability to inhibit α-glucosidase and pancreatic lipase. The IC50 values for saponin- and sapogenin-rich extracts inhibiting α-glucosidase were 0.10 and 2.98 mg/mL, respectively. Non-hydrolysed extracts also had a stronger inhibitory effect than acarbose. In the case of pancreatic lipase, only the hydrolysed extracts exhibited inhibitory effects on pancreatic lipase (IC_50_ of 7.60 mg/mL). Thus, *P. cambodiana* bark may be an applicable natural resource for preparing ingredients for functional products with inhibitory activity against α-glucosidase and pancreatic lipase. The phenolic contents, saponin contents, and antioxidant activities of the dried extract stored at a low temperature of 25 °C for 2 months showed the best stability, with more than 90% retention.

## 1. Introduction

*Pouteria cambodiana* (Pierre ex Dubard) Baehni, a perennial plant belonging to the Sapotaceae family, has a wide distribution across Asia [1]. In Thailand, it is commonly referred to as ‘Nom-nang’. Breastfeeding mothers in Thailand have traditionally consumed a daily oral decoction of the bark to promote lactation. *P. cambodiana* is one of the 325 species of the *Pouteria* genus, which has been reported to have immunomodulatory, antioxidant, and antihaemolytic activities. The methanol extract derived from the stem bark of *P. cambodiana* exhibits antioxidant properties, as evidenced by its IC50 value of 0.24 mg/mL against DPPH. In terms of pharmacological activities, extracts of *P. cambodiana* stem bark have been used for the treatment of inflammation, diabetes, indigestion, diarrhoea, nausea, and vomiting, and for the alleviation of back pain. The review by Fitriansyah et al. [2] showed that carotenoids, flavonoids, and phenolic compounds, including stilbenes, protocatechuic acid, and terpenoids, are the secondary metabolites that have been isolated from *P. cambodiana* extracts. Saponins are a class of specialised plant terpenoids that are derived from the mevalonic acid pathway. They represent the largest group of bioactive compounds that have been isolated from Pouteri [3]. 

There has been a growing interest in saponins in recent years. The pharmacological activities demonstrated by certain isolated saponins include hypocholesterolaemic, anticarcinogenic, antioxidant, hypoglycaemic, and antiprotozoal effects [4]. Saponins have been widely used in beverages and confectionaries, as well as in cosmetics. These compounds are composed of a non-polar sapogenin, either a triterpenoid or a steroid, which is attached to sugar moieties. They have been shown to have beneficial effects on lipid metabolism [5]. Hyperlipidaemia is a significant modifiable risk factor for various diseases, such as type II diabetes, cardiovascular diseases, cancer, obesity, osteoarthritis, asthma, and chronic back pain [5]. The treatment of these conditions sometimes necessitates the application of pharmaceuticals that restrict the absorption of fats and cholesterol in the intestines, such as orlistat and statins. These medications work by inhibiting the biosynthesis of cholesterol and promoting the excretion of bile acids in faecal matter [6]. Nevertheless, it is important to note that these drugs can potentially result in various adverse effects, such as constipation, osteoporosis, myopathy, oily stools, and dyspepsia [7]. Furthermore, diabetes mellitus is a chronic endocrine disorder characterised by elevated blood sugar levels resulting from inadequate production or utilisation of insulin, leading to disruptions in the metabolism of carbohydrates, fats, and proteins.

The presence of diabetes has been found to be associated with high levels of oxidative stress and a reduction in antioxidant capacity. One therapeutic strategy for managing diabetes involves mitigating the increase in postprandial blood glucose levels in diabetic patients. This is accomplished by hindering the absorption of sugar through the inhibition of gastrointestinal carbohydrate-degrading enzymes, especially α-glucosidase. Inhibitors of these enzymes interrupt the process of carbohydrate breakdown and extend the overall pace at which carbohydrates are digested. As a result, the rate of glucose absorption is slowed down, leading to a decrease in the postprandial plasma glucose increase. Therapeutic targeting of α-glucosidase has been identified as a potential intervention for managing postprandial hyperglycaemia, which represents the initial metabolic dysregulation observed in individuals with noninsulin-dependent diabetes mellitus (NIDDM) [8]. Certain α-glucosidase inhibitors, such as acarbose, miglitol, and voglibose, have been identified as effectively reducing the postprandial increase in blood glucose levels. These inhibitors primarily achieve this by inhibiting the activity of enzymes responsible for digesting carbohydrates and delaying the absorption of glucose. They have been found to be associated with various side effects, including abdominal distention, flatulence, meteorism, and potentially diarrhoea. Numerous traditional medicinal plants and plant-derived components have been documented as exhibiting inhibitory activity against α-glucosidase [9]. Therefore, the investigation of biologically active compounds derived from a wide range of plants remains a compelling approach in the search for inhibitors with diverse structures.

For this reason, the aims of this study were to (1) establish the best conditions for the ultrasound-assisted extraction (UAE) of saponins from *P. cambodiana* bark, (2) evaluate the in vitro lipase and α-glucosidase inhibitory potentials of saponin and steroid-type saponins (sapogenins)-rich extracted from *P. cambodiana*, and (3) prepare a saponin-enriched powder from the aqueous extract by using spray drying and then performing stability analysis of the powder during 3 months of storage.

## 2. Materials and Methods

### 2.1. Plant Material

Barks of *P. cambodiana* were purchased from the Community Enterprise Group in Maerim City, Chiang Mai, Thailand. As a pre-treatment, the bark was prepared by drying in a hot air oven (Memmert UF 110, Schwabach, Germany) at 60 °C for 48 h until the moisture content was lower than 10%. The dried barks were then ground into fine powder using a fine grinder equipped with a 200-mesh sieve. The powder was carefully packaged in a vacuum-sealed aluminium foil bag and stored in a cool and dry environment at a temperature ranging from 3 to 5 °C until further analysis. All compounds used in this study were of analytical grade.

### 2.2. Ultrasound-Assisted Extraction (UAE)

The process for the extraction of saponin from the *P. cambodiana* bark powder by UAE involved the use of ultrasonic probe equipment (VX500, Newtown, CT, USA) operating at 50% amplitude with a maximum power of 500 W and a frequency of 20 kHz [10]. The impact of various extraction temperatures (40, 50, and 60 °C) and ethanol concentration levels (50, 60, and 70%) on the saponin compound was assessed. Following the UAE process, the obtained extract was subjected to centrifugation at 5000 rpm for 15 min at 4 °C using a Nüve NF400R centrifuge (Ankara City, Turkey). This centrifugation step was performed to separate the supernatant from the residue. As a follow-up to the centrifugation process, filtration was performed using Whatman filter paper 41 to eliminate any remaining extremely tiny particles present in the supernatant. The extracted sample was concentrated using a rotary vacuum evaporator (Büchi, Straubenhardt, Germany) at 55 °C for 2 h. Subsequently, it was dried in a hot air oven at 55 °C for a period of 8–10 h. Following the drying process, the dried extract was stored at 4 °C for the purpose of conducting further analysis on the total phenolic, flavonoid, and saponin contents within the extracts.

### 2.3. Determination of Total Phenolic Contents (TPC)

A portion of the supernatant (approximately 1 mL) was diluted with water to a volume of approximately 7 mL. Subsequently, 0.5 mL of the Folin–Ciocalteau phenol reagent was introduced. A volume of 1.0 mL of a saturated solution of sodium carbonate was precisely added after a duration of 5 min. After that, the mixture was diluted with water until reaching a total volume of 10 mL. The samples were subjected to incubation at room temperature for 2 h. Following the incubation period, the absorbance at a wavelength of 765 nm was measured using a UV spectrophotometer (Agilent, Penang, Malaysia). The standard curve was generated using gallic acid, and the outcomes were expressed as mg of gallic acid equivalent per g of dry weight sample (mg GAE/g). The equation for the calibration curve was determined to be Y = 10.344X + 0.0486, where Y represents the light absorbance and X represents the compound concentration. Additionally, the determination coefficient (R^2^) was calculated to be 0.9973 [11].

### 2.4. Determination of Total Flavonoid Content (TFC)

Samples (0.25 mL) were mixed with 1.25 mL of distilled water and 75 µL of 5% NaNO_2_ solution and incubated for 6 min at room temperature. The mixture was then combined with 150 μL of 10% AlCl_3_ and 500 μL of 1 M NaOH and brought to 275 mL with distilled water. The solution was mixed thoroughly and left for 5 min at room temperature. Its absorbance was recorded using a spectrophotometer at 510 nm. The equation for the calibration curve was determined to be Y = 0.0153X + 0.2225, where Y represents the light absorbance and X represents the compound concentration. The determination coefficient (R^2^) was calculated to be 0.9938. Catechin equivalents per g of sample (mg CAE/g) were used to express total flavonoid contents [12].

### 2.5. Determination of Total Saponin Contents (TSC)

The quantification of total saponin was measured using the vanillin–perchloric acid method. This method relies on a colour reaction between the acid-hydrolysis products of saponin and vanillin. The crude extract powder, weighing 0.1 g, was dissolved in 10 mL of distilled water. A volume of 50 μL of the prepared sample solution was carefully transferred into a colorimetric tube. Subsequently, the solvent was evaporated at a controlled temperature of 60 °C using a water bath. A solution of vanillin with a concentration of 5.0% (0.2 mL) was freshly prepared and combined with 0.8 mL of perchloric acid with a concentration of 70% (*v*/*v*). The mixture was thoroughly mixed and incubated at 70 °C for 15 min. Following a 30-s immersion in an ice-water bath, a volume of 5 mL of glacial acetic acid was then introduced and thoroughly mixed. The quantification of total saponin contents was performed using spectrophotometry at a wavelength of 560 nm using a spectrophotometer (Hitachi, Model U-2001, Tokyo, Japan). Quantification was accomplished through the utilisation of a saponin calibration curve, represented by the equation Y = 0.1003X − 0.0123. The determination coefficient, denoted as R^2^, was determined to be 0.9934. In this context, Y represents light absorbance, while X represents the concentration of the compound. In order to calculate the average values of the respective accessions, the total saponin content in the extract was determined by conducting three replicates per sample in the experiment. The results were expressed as mg/g [13].

### 2.6. Preparation of the Saponin-Rich Extracts

The purpose of this procedure was to achieve a greater concentration of saponins in the extract samples obtained from the UAE. This is commonly accomplished through a liquid–liquid extraction method using aqueous butanol [14]. Therefore, following the identical procedure for obtaining the saponin-rich extracts, an additional concentration step was carried out using the method described by Chan et al. [15] with some modifications. In brief, following the acquisition of the crude extract, a mixture of 50 mL of water and 100 mL of n-butanol was added to the dried residue at a sample-to-solvent ratio of 1:20 (*w*/*v*). The resulting mixture was vortexed for 1 min and subsequently subjected to centrifugation at 5000 rpm for 10 min. The upper supernatant layer of n-butanol was filtered, while the lower layer was subsequently subjected to another extraction using water and butanol under identical conditions. The collected lower supernatants were dried using a rotary evaporator at 55 °C for 2 h, followed by further drying in a hot air oven at the same temperature for 30 min. The dried powder of saponin-rich extracts was quantified as TPC, TFC, and TSC. The dried extracts were stored at −20 °C until they were required for subsequent experimentation. The extractions were conducted with a minimum of two replicates.

### 2.7. Screening Process of Triterpenes and Steroids

The identification of any triterpenes and steroids present in the extract of *P. cambodiana* bark was performed using the Liebermann–Burchard test (LB test). Briefly, an aliquot of the extract was transferred into an evaporation dish and subjected to evaporation in a water bath until complete dryness was achieved. Subsequently, a small quantity of acetic anhydride was introduced into the solution, followed by gentle agitation using a glass rod. Then, a small amount of concentrated sulfuric acid was carefully added to the solution. The observation of a red-purple colour in the extract indicated the presence of triterpenes. The presence of steroidal structures could be inferred from the emergence of a greenish-blue colour. The coexistence of both colours within a layer indicated the presence of both triterpenes and steroids [16].

### 2.8. Preparation of the Sapogenin-Rich Extracts through Acid Hydrolysis of Saponins

The acid hydrolysis of previously obtained saponin-rich extracts was performed to produce sapogenin-rich extracts, following the methodology outlined by Navarro del Hierro et al. [17]. In brief, the saponin-rich extracts were subjected to a heat treatment at 100 °C in the presence of a HCl solution with a concentration of 2 mol/L. The ratio of sample to acid solution used was 1:50 (*w*/*v*), and the duration of the treatment was 1 h. After that, the mixture was ice-cooled for 5 min. It was then subjected to liquid–liquid extraction using ethyl acetate in a 1:1 (*v*/*v*) ratio. The extraction process involved vortex agitation for 1 min, followed by centrifugation at a speed of 5000 rpm for 5 min. The upper phase was collected, and the lower phase was extracted once more using an equal volume of ethyl acetate under the specified conditions. Both of the collected phases were dried using a nitrogen stream, and the resulting extract was stored at −20 °C until it was required for future use. This procedure was conducted at least in triplicate, and the final extracts were mixed to obtain a single final extract for further analysis.

### 2.9. α-Glucosidase Inhibition Assay Using Saponin and Sapogenin-Rich Extracts

The examined extract was first dissolved in DI water, while acarbose (Sigma-Aldrich, Darmstadt, Germany) was dissolved in phosphate buffer to provide five different concentrations ranging from 0.005 to 5 mg/mL. A tested extract was used to generate five distinct concentrations ranging from 0.001 to 10 mg/mL within the reaction mixture.

The determination of α-glucosidase enzyme (Sigma Aldrich, G5003-100UN) inhibition was conducted in a 100 mM sodium phosphate buffer with a pH of 6.8. The reaction involved the combination of 0.1 U/mL of the enzyme with 1.25 mM p-Nitrophenyl-α-D-glucopyranoside (pNPG) (Sigma-Aldrich, N1377-1G) in a final reaction volume of 200 μL at 37 °C. The extracts (10 μg/mL) were tested for their effect on enzyme activity in both the absence and presence of the extracts. The positive control used in this experiment involved the reaction in the presence of acarbose at a concentration of 10 μg/mL. A single unit of α-glucosidase activity was determined as the quantity of α-glucosidase enzyme needed to release one μmole of p-nitrophenol (pNP) from pNPG within a time span of 1 min. The reaction was initiated by the addition of pNPG. The released pNP was quantified by measuring the absorbance at 410 nm using a spectrophotometer (Fluostar Optima Microplate Reader, BMG Labtech, Offenburg, Germany) after a reaction time of 10 min. The elimination of background readings was achieved by subtracting the absorbance of the mixture in the absence of enzymes. The absorbance measurement was determined to be directly proportional to the enzymatic activity. The determination of α-glucosidase inhibition was carried out as follows:

(1) $$\mathrm{Inhibitory}\;\mathrm{activity}\;(\%)=\left(1-\frac{A}{B}\right)\times 100$$

where *A* represents the absorbance observed in the presence of the test substance, and *B* represents the absorbance observed in the control.

The inhibition assay, employing different concentrations of inhibitor, was also used to determine the concentrations of extracts that led to 50% inhibition (IC_50_ values) in comparison to acarbose. All measurements were conducted in triplicate [18].

### 2.10. Assay for Pancreatic Lipase Enzyme Inhibition Using Saponin and Sapogenin-Rich Extracts

The extract under examination was initially dissolved in deionized water, while orlistat was dissolved in a 10% solution of dimethylsulfoxide (DMSO). This process resulted in the production of five distinct concentrations ranging from 0.005 to 5 mg/mL. The activity of porcine pancreatic lipase (type II, from porcine pancreas) was quantified using a colorimetric assay. This assay measures the release of p-nitrophenol from Sigma (St. Louis, MO, USA) with minor modifications. The enzyme solutions were prepared just prior to use. The crude porcine PL type II (Sigma, EC 3.1.1.3) was suspended in a HCl buffer (2.5 mmol, pH 7.4, with 2.5 mmol NaCl) to achieve a concentration of 5 mg/mL (200 units/mL). The suspension was mixed using a stirrer for 15 min. The solution was subsequently subjected to centrifugation at 1500× *g* for 10 min, resulting in the retrieval of the clear supernatant. The pancreatic lipase solution (0.10 mL) was preincubated (Titramax 1000 package, Heidolph Instruments, Schwabach, Germany) with various concentrations (0.0001–10 mg/mL) of the extract and the selected compounds. The preincubation was carried out at 37 °C for 5 min. Subsequently, the p-nitrophenyl butyrate (pNPB) substrate (10 mM in acetonitrile) was added. The solution was then diluted to a volume of 1 mL using the *Tris*–HCl buffer prior to measuring the solution absorbance spectrophotometrically (Infinite M200, Tecan, Salzburg, Austria) at 410 nm. The absorbance was measured at a minimum of five time points: 1, 2, 3, 4, and 5 min. The release of pNP was determined by measuring the rise in absorbance at 410 nm relative to a blank sample using a denatured enzyme. The activity of pancreatic lipase is associated with the rate at which pNP is released. This rate can be approximated by analysing the slope of the linear segment observed in the absorbance vs. time profiles. The final concentration of DMSO was maintained at a fixed level and did not exceed 2.0%. The percentage of residual activity of pancreatic lipase for each compound was determined by comparing the lipase activity of PL with and without the compound. The assays were all performed in triplicate, resulting in inhibition percentages that represented the average of three observations. The assay mixture included orlistat, a well-established inhibitor of pancreatic lipase, which served as the positive control [19]. The inhibition activity was determined using the following formula:

(2) $$\mathrm{Inhibitory}\;\mathrm{activity}\;(\%)=\frac{\left[\left(A-B\right)-\left(C-D\right)\right]}{\left(A-B\right)}\times 100$$

where *A* represents the fluorescence observed when the enzyme and substrate were present but without the test substance (adding the stop solution after the enzymatic reaction); *B* represents the fluorescence observed when the enzyme and substrate were present but without the test substance (adding the stop solution before the enzymatic reaction); *C* represents the fluorescence with the presence of enzyme, substrate, and test substance (adding the stop solution after the enzymatic reaction); and *D* represents the fluorescence with the presence of enzyme, substrate, and test substance (adding the stop solution before the enzymatic reaction). The concentration of each sample was confirmed in triplicate (*p*-value < 0.01). The IC50 value denotes the concentration necessary to achieve a 50% inhibition of pancreatic lipase activity.

### 2.11. Storage Stability Test on the Saponin-Rich Extract Powder

A 10% (*w*/*v*) concentration of maltodextrin was added to the saponin-rich extracts. The mixture was then homogenised using a high-pressure homogeniser (IKA scientifica, Königswinter, Germany) at a pressure of 10,000 psi. The spray-drying process was conducted using a laboratory-scale spray dryer (Büchi, Flawil, Switzerland). The liquid feed rate was set at 0.6 L/h, while the spray air flow rate was maintained at 0.5 m^3^/h. The aspirator flow rate for drying air was fixed at 80%. The inlet air temperature was set to 140 °C, while the outlet temperature was maintained at 80 ± 5 °C. The microcapsules were transferred to aluminium foil bags and subsequently sealed under vacuum conditions prior to being stored at controlled temperatures of 25, 35, and 45°C. The stability of the extract powder was assessed at regular one-month intervals for three months by analysing its moisture content, water activity, colour, TPC, TSC, and antioxidant activity. Every test was conducted in triplicate.

### 2.12. Antioxidant Analysis

#### 2.12.1. DPPH Radical Scavenging Capacity Assay

The capacity of the saponin-rich extract powder to effectively scavenge DPPH (2,2-diphenyl-1-picryl-hydrazyl radical) was evaluated using the methodology outlined in the study conducted by Lu et al. [20]. The measurement of absorbance at a wavelength of 517 nm was conducted using a UV spectrophotometer (Thermo Fisher Scientific, Waltham, MA, USA). The calibration curve was established using Trolox, and the measurements were expressed as µmol of Trolox per g of dry weight sample (µM Trolox/g). The equation representing the calibration curve can be expressed as Y = −0.0153X − 0.6391. Additionally, the determination coefficient, denoted as R^2^, was calculated to be 0.9911. In this context, Y represents the light absorbance, while X represents the concentration of the compound.

#### 2.12.2. Ferric-Reducing Antioxidant Power (FRAP) Assay

The FRAP analysis of the saponin-rich extract powder was conducted using the methodology outlined in the study by Sharma et al. [21]. The measurement of absorbance at a wavelength of 593 nm was performed using a UV spectrophotometer (Thermo Fisher Scientific, MA, USA). The compound Trolox was employed in the development of a calibration curve, and the results were expressed as µmol of Trolox equivalents per g of dry weight sample (µM Trolox/g). The equation representing the calibration curve was Y = 0.0043X + 0.0186. The determination coefficient, denoted as R^2^, was calculated to be 0.997. In this context, Y represents the light absorbance, and X represents the compound concentration.

### 2.13. Moisture Content and Water Activity

The moisture content of the samples was determined by subjecting them to a drying process in an air oven at 70 °C for 24 h, in accordance with the guidelines set by Turkish standards (TSE, 2000). Water activity measurements were conducted at 25 °C using an AquaLab water activity metre (4TE, Decagon Devices, Inc., Pullman, WA, USA). 

### 2.14. Colour Determination

The colour parameters of the samples were measured using a colour meter (Konica Minolta, Tokyo, Japan) and reported as L*, a*, and b* values. The L* value denotes the level of lightness, with 100 indicating maximum lightness and 0 indicating maximum darkness. The a* value represents the presence of red (+) or green (−) chromaticity, while the b* value represents the presence of yellow (+) or blue (−) chromaticity.

### 2.15. Statistical Analysis

All data were expressed as the mean ± standard deviation (SD). The data were analysed using a one-way analysis of variance (ANOVA), followed by Tukey’s post hoc test for multiple comparisons using Statgraphics 5.1 Software. A *p*-value of <0.05 was deemed statistically significant.

## 3. Results and Discussion

### 3.1. Influence of UAE Parameters on the Extraction Efficiency

The role of temperature is of significant importance in the process of saponin extraction. In order to mitigate the degradation of thermolabile compounds, it is imperative to select the extraction temperature based on the specific components being targeted for extraction. Table 1 displays the quantities of extracted saponins observed at various temperatures (40, 50, and 60 °C). A consistent increase in content was noted upon the temperature reaching 50 °C. The saponin content reached its maximum value of 36.04 ± 0.08 mg/g at 50 °C. This temperature facilitated the solubility of the saponins and enhanced the molecular movement of the solute. The saponin content at all levels of ethanol concentration also increased when the temperature increased from 40 to 50 °C. The elevation of the temperature has several effects on the extraction process. Firstly, it enhances the solubility of compounds, making them more easily dissolved in the extraction medium. Additionally, it reduces the viscosity and surface tension of the medium, which aids in the extraction process. Furthermore, the increase in temperature facilitates molecular collisions at the interface of the mixture, promoting efficient mass transfer. Lastly, it induces an increase in vapour pressure, which can have implications for the extraction process [22]. The latter phenomenon results in the vapour-filled bubbles experiencing a cushioning effect during cavitation, meaning that the intensity of cavitation is inversely proportional to temperature [23]. Nevertheless, it has been observed that an excessively high extraction temperature (60 °C) may adversely impact the extraction efficiency of saponins. This could be attributed to the potential decomposition and denaturation of saponins, ultimately resulting in a decrease in the overall saponin content [24]. Anh et al. [25] also reported comparable findings. In order to optimise the extraction yield and effectively manage energy consumption, a temperature of 50 °C was chosen for subsequent experiments. The observed increase in total phenolic content from temperature variations was also found to be associated with the total saponin content.

The concentration of the solvent is another significant parameter to consider when extracting saponins. Upon analysis of the experimental data presented in Table 1, it was evident that the use of 70% ethanol as a solvent resulted in the highest total saponin contents, ranging from 31.94 to 35.69 mg/g. The total saponin content exhibited a steady increase as the ethanol concentration was raised from 50% to 70%. The highest concentration level was observed at 70% ethanol for both the temperature levels of 40 and 60 °C. Very similar results have been obtained in total phenolic and flavonoid content. Upon contact with the plant material, the solvent will cover it, initiating a gradual swelling process. Hydrogen bonds are formed between the solvent (ethanol) and the hydroxyl groups present in the cellulosic structure of the plant material. This outer layer of solvent can impede the efficiency of extraction by obstructing the diffusion process. It contains a high concentration of extracted compounds derived from the plant. Ultrasound is utilised to disrupt hydrogen bonds and facilitate the removal of the superficial layer through the generation of high-speed jets resulting from the collapse of asymmetrical bubbles, and this process enhances the diffusion process [26]. Nevertheless, when the ethanol concentration was increased at an extraction temperature of 50 °C, there was no statistically significant difference (*p* > 0.05) observed in the total saponin content at 60 and 70%. Based on the obtained results of the highest content of saponin and considering the economic perspective of using smaller quantities of organic solvent and minimising energy consumption, the extraction temperature of 50 °C and an ethanol concentration of 50% were determined to be the optimal conditions for this experiment. This optimal extraction condition for saponin was selected for further preparation of the saponin-rich extract. The purpose of this procedure was to achieve a greater concentration of saponin in the extract samples obtained from the UAE.

The crude extracts of *P. cambodiana* from the UAE procedure have complex ingredients such as saponins, phenolic compounds, flavonoids, sterols, and volatile compounds. Therefore, the separation of the crude extracts for saponin-rich extract preparation was essential to assess the effect on α-glucosidase and pancreatic lipase inhibition. From Table 2, the separation procedure through a liquid–liquid extraction method using aqueous butanol achieved a greater concentration of saponins (283.3 ± 0.93 mg/g) in the extract samples obtained from the UAE. This was accomplished to improve the relatively high initial saponin concentration and eliminate other compounds which showed the lower content of TPC (60.71 ± 0.20 mg GAE/g sample) than the previous crude extract sample. Hence, the separation of saponins from the crude extracts was important to improve saponin purity.

### 3.2. Comparative Effect of Non-Hydrolysed and Hydrolysed Extracts on α-Glucosidase Inhibition

Polyphenols found in plants possess antioxidant properties and have been observed to exhibit antihyperglycemic effects. These effects are believed to be a result of their non-specific binding to glucose transporters and competitive inhibition of digestive enzymes [27]. The α-glucosidase enzyme is located within the brush border of the epithelium in the small intestine. It facilitates the hydrolysis of disaccharides and starch into glucose [28]. Glucosidase inhibitors have the effect of reducing the rate of carbohydrate digestion and slowing down the absorption of carbohydrates from the alimentary tract [29]. Extensive research is currently being conducted to identify phytoconstituents with potential hypoglycaemic properties, particularly those that can inhibit α-glucosidase. The current study was intended to discover effective hypoglycaemic agents derived from natural sources.

**Figure 1:**
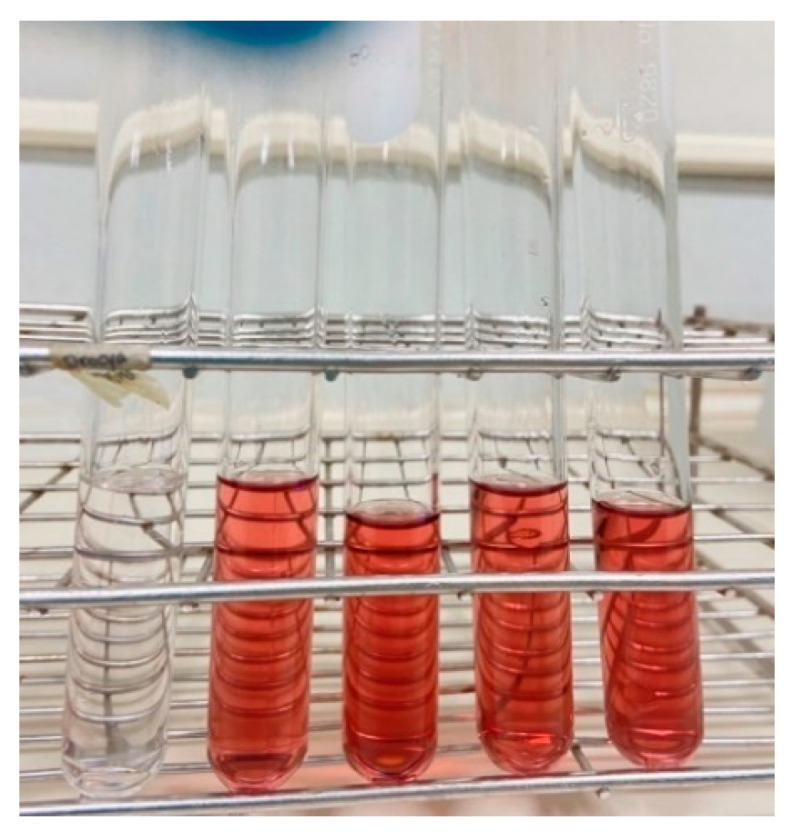
Triterpenoid saponin identification through the Liebermann–Burchard test.

The evaluation of the inhibitory effects of saponin-rich extracts against α-glucosidase was conducted, and the results were compared with those of acarbose. These findings are presented in Table 3. The saponin-rich extracts demonstrated strong inhibition of α-glucosidase, with IC50 values of 0.10 ± 0.01 mg/mL. This accords with Silva et al. [30], who illustrated the inhibitory effect of various concentrations of crude extracts derived from *P. cambodiana* bark on α-glucosidase. Traditionally, saponins are classified into triterpenoids and steroids on the basis of the aglycone skeleton. However, the colour shaded test on *P. cambodiana* extract revealed the presence of triterpenoid saponins in the butanol extract. This was evident from the appearance of a red-purple colour during the LB test, as presented in Figure 1. Therefore, the main constituents of the genus *Pouteria*, which is classified under the family Sapotaceae, are triterpenes and flavonoids. The review by Mohamed Abed El Aziz et al. [31] showed that triterpenoid saponins are surface-active glycosides of triterpenes that possess a wide, biologically active group of terpenoids and include a large chemical diversity of secondary metabolites with more than 100 different carbon skeletons. A previous study by Li et al. [32] reported that triterpenoid saponins from *Aralia taibaiensis* (Araliaceae) have an inhibitory effect on α-glucosidase. They speculated that the main active ingredients were triterpenoid saponins that have the C-3 position in the active ingredient structure, such as the β-D-glucopyranosyl-(1→2)-[β-D-glucopyranosyl-(1→3)]-β-D-glucuronopyranosyl oligosaccharide moiety. Dou et al. [33] also found that the presence of the glucuronic acid unit at C-3 and glucosidic moiety at C-28 of the aglycone were crucial functional groups for inhibiting α-glucosidase activity. They observed that substituting or adding a different sugar unit in place of glucose could potentially eliminate the inhibitory effect.

As shown in the present study, there is a direct correlation between the increase in concentrations and the corresponding increase in the inhibition rate of α-glucosidase. The saponin-rich extracts exhibited a higher IC50 value of 0.10 ± 0.01 mg/mL, while acarbose demonstrated a slightly lower IC50 value of 0.07 ± 0.01 mg/mL. However, this difference in inhibition of α-glucosidase was not found to be statistically significant (*p* ≥ 0.05). Our findings align with the research conducted by Fu et al. [34], which supported previous reports on the inhibitory effect of triterpenoid saponins on α-glucosidase activity. Additionally, research indicates that the structural components of flavonoids and triterpenoid saponins play a crucial role in their ability to inhibit α-glucosidase. This includes the positioning of sugar moieties in the aglycon of triterpenoid saponins. Hence, it can be inferred that the extracts derived from *P. cambodiana* bark exhibit promising inhibitory properties against α-glucosidase. The primary constituents responsible for these effects are likely triterpenoid saponins. However, in future research endeavours, we intend to conduct additional in vivo assays to further validate the potential hypoglycaemic activity of the extracts. 

**Figure 2:**
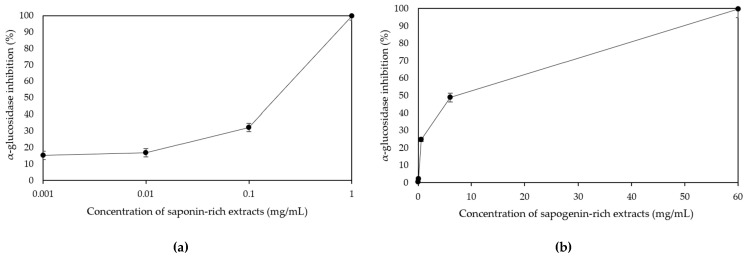
Inhibitory effect on α-glucosidase of different concentration levels of saponin- (**a**) and sapogenin-rich extracts (**b**).

The chemical structure of saponins can undergo transformation through acid hydrolysis, leading to the generation of aglycones, prosapogenins (partially hydrolysed saponins), and sugar residues. Sapogenin-rich extracts were obtained in the present study by acid hydrolysis and followed by an extraction of sapogenins with ethyl acetate. The final dried extracts showed a sapogenin content of 22.71 mg/g. The effect of the hydrolysis of the saponin-rich extracts on the activity of α-glucosidase inhibition was also evaluated. According to Figure 2, both non-hydrolysed and hydrolysed extracts demonstrated the capacity to inhibit pancreatic α-glucosidase activity in a dose-dependent manner. This enabled the estimation of their IC50 values, as presented in Table 3. When examining each of the extracts individually in Table 3, it was observed that the non-hydrolysed extracts displayed the lowest IC50 value (0.10 ± 0.01 mg/mL). This value was significantly lower (*p <* 0.05) compared to the IC50 values of the hydrolysed extracts (2.98 ± 0.33 mg/mL) and acarbose (0.15 ± 0.01 mg/mL). In other words, the non-hydrolysed extracts exhibited greater efficacy in inhibiting α-glucosidase activity compared to both the hydrolysed extracts and acarbose. 

The triterpenoid saponins demonstrated a strong binding affinity for α-glucosidase. The potential binding of amino acid residues in the catalytic active site of α-glucosidase could occur through hydrogen bonds and hydrophobic interactions. These interactions may involve specific amino acid residues, including His280, Gly309, Asn 247, Ser240, Asp307, Pro312, Leu246, Val319, Val308, and Ala329. The saponin-rich extracts also exhibited the presence of glucuronic acid groups at the C-3 position, which were observed to form hydrogen bonds with the amino acid residue. According to Su et al. [35], it is also postulated that hydrogen bonds play a significant role in suppressing catalytic activity. Additionally, the presence of a carboxyl group at the C-28 position has the potential to form hydrogen bonds, leading to conformational changes in α-glucosidase. This, in turn, may result in a reduction in its catalytic activity [36]. In addition, the aglycones located at position C-28, which are connected to the glucose component, have demonstrated significant inhibitory effects on the activity of α-glucosidase [33]. The inhibitory effect on α-glucosidase is primarily influenced by hydrogen bonds and hydrophobic interactions. These interactions are mainly determined by the characteristics of the residues involved in the interaction and the compounds themselves [37]. Saponins exhibit amphipathic properties due to their composition, which consists of a hydrophilic sugar moiety, referred to as a glycoside or glycone, and a lipophilic steroidal moiety, known as a sapogenin or aglycone. These two components are connected through an O-linked glycosidic bond. The acid hydrolysis process results in the conversion of saponin, causing the dispersion of glycoside in an aqueous solution. Hence, the absence of glycoside leads to a reduced ability of the precipitated sapogenin to enhance α-glucosidase inhibition [38].

### 3.3. Effects of Non-Hydrolysed and Hydrolysed P. cambodiana Bark Extract on Pancreatic Lipase Activity

Pancreatic lipase plays a crucial role in the absorption of dietary triacylglycerol by catalysing the hydrolysis of triacylglycerol into 2-monoacylglycerol and fatty acids. It is widely recognised that dietary fat cannot be directly absorbed from the intestine unless it has undergone the enzymatic action of pancreatic lipase [39]. The findings indicated that the inhibitory effects of *P. cambodiana* bark extract on pancreatic lipase varied among the saponin- and sapogenin-rich extracts, and orlistat. The sapogenin-rich extracts demonstrated an IC50 value of 7.60 ± 0.01 mg/mL, which was significantly lower compared to the saponin-rich extracts (˃1000 mg/mL). Among the treatments evaluated, it was observed that both the orlistat and the sapogenin-rich extracts exhibited significant inhibitory effects against pancreatic lipase. However, the saponin-rich extract did not demonstrate similar inhibitory effects, as indicated in Table 4.

**Figure 3:**
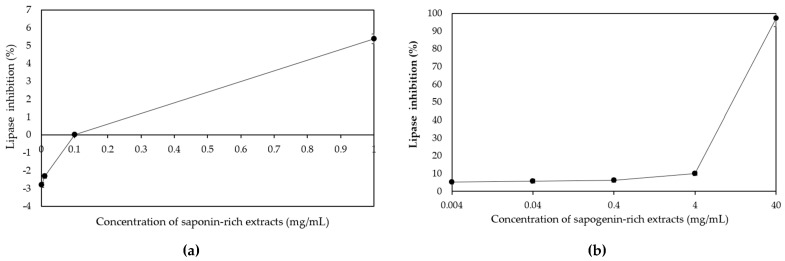
Inhibitory effect on pancreatic lipase of different concentration levels of saponin- (**a**) and sapogenin-rich extracts (**b**).

The hydrolysis of *P. cambodiana* bark extract has been found to enhance the inhibitory activity of pancreatic lipase (Figure 3). The majority of sapogenins have exhibited enhanced bioavailability, and certain bioactivities of sapogenins are even more prominent compared to their previous saponin forms. This can be attributed to the more favourable chemical properties resulting from the absence of the sugar chain [40]. Zhao et al. [41] conducted the hydrolysis of bidesmoside saponins derived from a methanolic extract of *Platycodi radix* to obtain prosapogenin, a triterpenoid glucoside that features a solitary sugar attached to C-3. The modified compound demonstrated a significant 30% enhancement in its ability to inhibit pancreatic lipase when compared to the previous saponins. The deglycosylation of triterpenoid glucosides, which involves the removal of a single glucose moiety, has been found to result in an increased inhibition of lipase activity [42]. In a study conducted by Navarro del Hierro et al. [43], it was demonstrated that a quinoa extract displayed the highest IC50 value (1.15 ± 0.15 mg/mL), which was found to be significantly greater (*p* = 0.001) than the IC50 value of the hydrolysed quinoa extract (0.74 ± 0.09 mg/mL). Similarly, Uemura et al. [44] also discovered that a hydrolysed fenugreek extract exhibited significant inhibition of triglyceride accumulation in HepG2 cells. Conversely, no effect was observed in the saponin fraction. One potential approach to enhance the specific bioactivities of saponins and discover novel bioactivities is through the conversion of saponins into sapogenins. Hence, the investigation into the inhibitory lipase activity of hydrolysed saponin-rich extracts, as exemplified by our recent findings on *P. cambodiana* bark extract, presents a compelling avenue for exploring novel natural products with such potential.

### 3.4. Effect of Storage Temperature on the Stability of Saponin-Rich Extract Powder

The initial moisture content and water activity of the saponin-rich extract powder were determined to be 5.26 ± 0.21% and 0.43 ± 0.21, respectively. According to the data presented in Table 5, the moisture content of the samples that were subjected to vacuum packaging exhibited an approximate increase of 1% after a storage period of 2 months across all temperature levels. However, the moisture content of the samples remained consistent throughout the 2 to 3 months of storage. Based on the obtained results, it is evident that the temperature exerted an influence on the variations in the moisture content and water activity of the samples throughout the storage period. The extract stored at a higher temperature of 45 °C demonstrated a decrease in moisture content and water activity in comparison to the extract stored at lower temperatures of 25 and 35 °C. The elevation of the storage temperature expedited the decrease in moisture content of the powder extract. The findings of this study align with the observations reported by Forsido et al. [45], who found that food flour (specifically oats, soybeans, and linseed) stored in polyethene bags exhibited higher moisture content at low temperatures (−18 °C) compared to high temperatures (45 °C). Furthermore, the lowest water activity (0.18) was observed in samples that were stored for 90 days at 45 °C. According to Table 3, the water activity values of powder samples stored in aluminium foil remained consistently stable over a period of 3 months.

There was no statistically significant difference observed in the L* value results for the saponin-rich extract powder between the first and second months at temperatures of 25, 35, and 45 °C. However, there was a slight increase in the brightness of the powder observed during the third month of storage. There was no significant difference (*p* ≥ 0.05) in the a* and b* values at all storage temperatures until the third month. The a* values ranged from 7.67 to 8.13, while the b* values ranged from 14.23 to 14.72. Overall, the storage of samples at various temperature levels did not yield a notable alteration in colour.

The results indicated that the storage temperature had a significant effect on TPC and TSC, as shown in Table 5. Notably, variations were observed over the entire 3 month storage period. The TPC and TSC of the dried extract, which was stored at a low temperature of 25 °C for 2 months, exhibited optimal stability with a retention rate exceeding 90%. After a storage period of 3 months at a temperature of 45 °C, the extracts exhibited the lowest content values at 202.06 ± 0.72 mg GAE/g sample for TPC and 7.29 ± 0.06 mg/g sample for TSC. Regarding the statistical analysis, it was determined that both temperature and time had a significant effect (*p* < 0.05) on TPC and TSC. According to the data presented in Table 3, it can be observed that the antioxidant capacity of saponin-rich extract powder derived from *P. cambodiana* bark was significantly affected by both the storage temperature and time. Furthermore, the alterations in DPPH and FRAP exhibited comparable patterns. The levels of both DPPH and FRAP showed a general decrease over the course of the 3-month storage period. However, there was only a slight reduction observed after 2 months of storage, followed by a significant decrease (*p* < 0.05) in the last month. After a storage period of 3 months at 45 °C, the levels of DPPH and FRAP exhibited the greatest decrease, resulting in values of 120.38 ± 2.53 and 79.48 ± 1.56 mg Trolox/g sample, respectively. The findings from the assessment of antioxidant activities suggested that a decrease in storage temperature may help alleviate the decline in DPPH and FRAP scavenging activity. The findings align with the study conducted by Kim et al. [46], which reported a similar trend. Specifically, their research indicated that the antioxidant activity of catechins derived from matcha (*Camellia sinensis*) exhibited a greater decline at elevated storage temperatures.

## 4. Conclusions

The application of ultrasound-assisted extraction (UAE) was assessed as an innovative method to optimise the efficiency of extracting triterpenoid saponins from *P. cambodiana* bark. The most favourable parameters for the UAE were an extraction temperature of 50 °C and an ethanol concentration level of 50%. Furthermore, this study provided support for the significance of saponin-rich extracts derived from *P. cambodiana* bark as a medicinal plant in the treatment of diabetes as well as a potential source of α-glucosidase inhibitors with promising therapeutic properties. Moreover, the hydrolysed extracts were found to contain sapogenins that exhibited inhibitory effects on pancreatic lipase, thereby enhancing the bioactivity of the non-hydrolysed extract. These findings suggest that these extracts have the potential to be utilised as a natural ingredient in functional foods aimed at preventing and managing postprandial blood glucose levels and diseases associated with hyperlipidaemia. The isolation of the identified compounds may contribute to the identification of novel, safe, and effective pharmaceutical agents.

## Figures and Tables

**Table 1:** The effects of extraction temperature and ethanol concentration on total saponin content derived from the barks of *P. cambodiana*.

Temperature (°C)	Ethanol Concentration (%)	Total Phenolic Content (mg GAE/g Sample)	Total Flavonoid Content (mg CAE/g Sample)	Saponin Content (mg/g)
40	50	234.9 ± 0.89 ^g^	59.76 ± 0.95 ^e^	20.23 ± 0.48 ^f^
	60	283.5 ± 0.55 ^d^	70.88 ± 0.14 ^c^	31.70 ± 0.70 ^c^
	70	308.9 ± 1.89 ^b^	76.63 ± 0.91 ^b^	31.94 ± 0.18 ^c^
50	50	314.7 ± 0.55 ^a^	79.85 ± 0.64 ^a^	36.04 ± 0.08 ^a^
	60	313.8 ± 0.36 ^a^	79.12 ± 0.61 ^a^	35.64 ± 0.09 ^a^
	70	313.6 ± 1.15 ^a^	80.43 ± 0.85 ^a^	35.69 ± 0.09 ^a^
60	50	246.5 ± 0.95 ^f^	63.76 ± 0.38 ^d^	26.57 ± 0.09 ^e^
	60	272.4 ± 1.09 ^e^	68.11 ± 0.27 ^c^	29.27 ± 0.14 ^d^
	70	294.4 ± 0.62 ^c^	73.00 ± 0.69 ^bc^	33.08 ± 0.61 ^b^

Note: Each value was expressed as the mean ± SD (*n* = 3). The presence of different superscript letters within a shared column denotes statistically significant differences (*p* < 0.05).

**Table 2:** Bioactive compounds of saponin-rich extract powder from *P. cambodiana* extracts.

Bioactive Compounds	Saponin-Rich Extract Powder
Total phenolic content (mg GAE/g sample)	60.71 ± 0.20
Total flavonoid content (mg CAE/g sample)	136.6 ± 0.45
Saponin content (mg/g)	283.3 ± 0.93

**Table 3:** Inhibitory effect on α-glucosidase (IC50) of saponin- and sapogenin-rich extracts of *P. cambodiana* bark compared to acarbose.

Samples	IC50 (mg/mL)
Acarbose	0.15 ± 0.01
Saponin-rich extracts	0.10 ± 0.01
Sapogenin-rich extracts	2.98 ± 0.33

Acarbose was used as a reference compound. The IC50 value represents the concentration required to inhibit 50% of the α-glucosidase activity.

**Table 4:** Inhibitory activities of *P. cambodiana* bark extracts on pancreatic lipase.

Samples	IC50 (mg/mL)
Orlistat	2.36 ± 0.02
Saponin-rich extracts	˃1000
Sapogenin-rich extracts	7.60 ± 0.01

Orlistat was used as a reference compound. The IC50 value represents the concentration required to inhibit 50% of the pancreatic lipase activity.

**Table 5:** Moisture content, water activity, color difference (L*, a*, and b*), total phenolic content, total saponin content, and antioxidant activities during storage of saponin-rich extract powder from *P. cambodiana* bark.

Temperature (°C)	Time (Months)	Moisture Content (% db.)	Water Activity	L*	a*	b*	TPC (mg GAE/g)	TSC (mg/g)	DPPH (mg Trolox/g)	FRAP (mg Trolox/g)
Control	0	5.26 ± 0.21 ^ef^	0.43 ± 0.00 ^a^	79.15 ± 0.34 ^ab^	8.06 ± 0.10 ^ab^	14.65 ± 0.18 ^ns^	241.42 ± 1.51 ^a^	10.19 ± 0.15 ^a^	262.34 ± 5.70 ^a^	131.73 ± 3.28 ^a^
25	1	5.93 ± 0.33 ^d^	0.43 ± 0.00 ^a^	79.69 ± 0.13 ^ab^	7.91 ± 0.05 ^ab^	14.27 ± 0.03 ^ns^	227.03 ± 0.10 ^b^	10.00 ± 0.07 ^a^	174.71 ± 3.16 ^b^	124.03 ± 1.12 ^b^
	2	7.68 ± 0.12 ^a^	0.43 ± 0.00 ^a^	78.71 ± 0.22 ^bc^	7.88 ± 0.25 ^ab^	14.27 ± 0.53 ^ns^	227.20 ± 1.54 ^b^	9.53 ± 0.30 ^b^	161.23 ± 3.29 ^c^	112.84 ± 1.33 ^e^
	3	7.19 ± 0.03 ^b^	0.44 ± 0.00 ^a^	79.29 ± 0.90 ^ab^	8.13 ± 0.28 ^a^	14.66 ± 0.31 ^ns^	214.29 ± 1.08 ^e^	8.65 ± 0.49 ^c^	133.25 ± 3.53 ^f^	95.76 ± 0.24 ^h^
35	1	5.88 ± 0.08 ^d^	0.43 ± 0.00 ^a^	78.59 ± 0.32 ^bc^	7.67 ± 0.27 ^b^	14.72 ± 0.25 ^ns^	222.35 ± 1.59 ^c^	8.56 ± 0.20 ^cd^	165.32 ± 2.15 ^c^	121.12 ± 0.76 ^c^
	2	6.93 ± 0.05 ^bc^	0.44 ± 0.00 ^b^	78.12 ± 0.57 ^cd^	7.92 ± 0.25 ^ab^	14.45 ± 0.35 ^ns^	219.75 ± 0.82 ^d^	8.45 ± 0.21 ^cd^	149.80 ± 3.29 ^d^	109.13 ± 1.23 ^f^
	3	6.67 ± 0.01 ^c^	0.42 ± 0.00 ^c^	79.75 ± 0.25 ^a^	7.85 ± 0.20 ^ab^	14.57 ± 0.17 ^ns^	207.00 ± 1.51 ^f^	8.26 ± 0.03 ^cd^	125.49 ± 2.36 ^g^	84.13 ± 0.63 ^i^
45	1	4.92 ± 0.07 ^f^	0.37 ± 0.01 ^d^	78.49 ± 0.23 ^cd^	7.81 ± 0.25 ^ab^	14.23 ± 0.36 ^ns^	218.62 ± 0.63 ^d^	8.55 ± 0.14 ^cd^	143.67 ± 2.25 ^e^	115.89 ± 1.29 ^d^
	2	5.53 ± 0.18 ^e^	0.39 ± 0.00 ^d^	77.71 ± 0.49 ^d^	7.92 ± 0.60 ^ab^	14.65 ± 0.23 ^ns^	215.85 ± 0.75 ^e^	7.29 ± 0.14 ^e^	141.01 ± 1.94 ^e^	105.57 ± 239 ^g^
	3	5.27 ± 0.04 ^ef^	0.36 ± 0.06 ^d^	79.03 ± 0.88 ^abc^	7.99 ± 0.18 ^ab^	14.68 ± 0.03 ^ns^	202.06 ± 0.72 ^g^	7.29 ± 0.06 ^e^	120.38 ± 2.53 ^h^	79.48 ± 1.56 ^j^

The data are expressed as the means ± standard deviation of three independent replicates (*n* = 3). Different superscript letters in the same column indicate a significant difference (*p* < 0.05).

## Data Availability

Data is contained within the article.
